# Progress to Date on Cranial Electromagnetic Field Stimulation to Modulate Brain Activity

**DOI:** 10.7759/cureus.84653

**Published:** 2025-05-22

**Authors:** Alice S Wang, Paras Savla, James Brazdzionis, Katherine Ko, Dan E Miulli

**Affiliations:** 1 Neurosurgery, Riverside University Health System Medical Center, Moreno Valley, USA; 2 Neurosurgery, Arrowhead Regional Medical Center, Colton, USA

**Keywords:** duration of treatment, electromagnetic field frequency, electromagnetic field stimulation, head trauma, voltage

## Abstract

Background: The electromagnetic field (EMF) of the brain can be modulated through EMF stimulation. The authors investigate whether longer duration of continuous EMF stimulation using a novel method to identify and provide feedback and adjustment of EMF recording would translate into sustained improvement in EMF patterns, such as higher amplitude with correlating improvement in clinical symptoms or deficits.

Methods: From January 2025 to February 2025, a prospective study enrolled patients greater than 18 years old diagnosed with atraumatic and traumatic brain injury who underwent EMF stimulation within 24 hours of presentation. EMF data were collected using DAQami software (Dataq Instruments, Akron, Ohio) and analyzed using fast Fourier transformation (FFT) with Igor Pro 8 software (Wavemetrics Inc., Lake Oswego, Oregon). Based on each patient’s clinical presentations and/or radiographic findings, localization of brain injuries, frequency selection, and optimal voltage stimulation were determined in real-time followed by delivery of incremental increase in duration of stimulation from 3, 5, 8, and 10 minutes until improvement in clinical symptoms and/or neurological deficits and sustained EMF change was achieved.

Results: Ten patients were included in this study, with a mean age of 47.1 years. Mechanisms of injury included spontaneous hypertensive intracranial hemorrhage (1 patient) and head trauma after motor vehicle collision, dirt bike accident, and ground-level fall (9 patients). Radiographic findings included spontaneous basal ganglia hemorrhage (1 patient), isolated traumatic subdural hematoma (1 patient), traumatic subarachnoid hemorrhage (1 patient), and no intracranial abnormalities (7 patients). Clinical resolution of their neurological symptoms or remaining asymptomatic was achieved in five patients after three minutes of continuous EMF stimulation, two patients after five minutes of continuous EMF stimulation, and one patient after 10 minutes of continuous EMF stimulation (Table 1). Patient 8 declined to continue with the study after three minutes of continuous EMF stimulation, and patient 9 declined to continue with the study after five minutes of continuous EMF stimulation.

Conclusions: This study reveals the progress made to date utilizing a novel technology of EMF measurement at a distance, in real-time, using the non-invasive, lightweight portable helmet, and continuous feedback. The range of brain EMF can be stimulated at the optimal frequency and voltage with or without longer duration of stimulation in a precise and prescribed manner to produce sustained genetic and neuronal changes to improve, recover, and enhance the brain function in a sample of patients with atraumatic and traumatic brain injury and improve or resolve their neurological symptoms or deficits. It illustrates the necessity of real-time evaluation and adjustment of brain EMF for EMF stimulation. It further indicates the efficacy of tailored and precise EMF stimulation to the specific patient, the specific area of abnormality, and for a specific pathology studied. The range of unique EMF corresponds to macroscopic and microscopic functions, the vast majority of which have yet to be qualified and quantified, and for which most brain diseases have yet to be studied.

## Introduction

Magnetoencephalography (MEG) measures the brain’s electromagnetic fields. Its non-invasive sensors map brain function and locate functional and abnormal activity. The electrochemical activities of the brain can be modulated through electromagnetic field (EMF) stimulation. The therapeutic effects of pulsed electromagnetic field (PEMF) and applied extremely low frequency magnetic fields (ELF-MF) in animals and humans demonstrate their utility in restoring brain functions. Studies have found PEMF and ELF-MF to provide neuroprotection and reduction in functional deficits in acute ischemic stroke, with underlying mechanisms involving extensive molecular, cellular, and neuronal changes [1-8]. In Capone et al., patients with acute ischemic stroke underwent pulsed EMF stimulated for 45 minutes per day or 120 minutes per day for 5 days total and clinical assessments (National Institute of Health Stroke Scale, Barthel Index, and modified Rankin Scale) were performed at 5 days, 1 month, 3 months, and 12 months. All six patients showed clinical improvements [6].

The therapeutic effects of continuous EMF stimulation with real-time clinical and EMF assessment have been studied [9]. Real-time recording and feedback provide guidance on the next step in EMF stimulation and facilitate maximally sustained restoration of brain activities. Here, the authors attempt using a novel non-invasive, portable helmet equipped with 20 sensors to both record normal and abnormal brain EMF, diagnose abnormal EMF, and adjust EMF stimulation parameters, specifically the duration of stimulation, based on real-time feedback from patients to achieve optimal change in EMF patterns and desired clinical improvement in patients with traumatic and atraumatic brain conditions [9-13]. The authors hypothesize longer duration of continuous stimulation when patients have non-sustained and non-optimal improvement on EMF measurements would translate into higher amplitudes on the EMF recording at the stimulated frequency of interest, with correlating improvement in sustained clinical symptoms or deficits.

## Materials and methods

Study design

Our institution’s institutional review board approved this prospective study (Arrowhead Regional Medical Center, Protocol #23-58). Inclusion criteria included patients greater than 18 years old with head trauma or intracranial hematomas. Exclusion criteria were a Glasgow Coma Scale (GCS) of 3, contraindications for donning a helmet, such as active hemodynamic or respiratory instability, or refusal of study enrollment. Two portable racks equipped with horizontal rods and four cords were used to suspend the helmet in the air, and the cords provided adjustable tension to securely hang the helmet in place just above the patient’s head. A portable lightweight helmet with shielding constrained to a dual-layered Mu-metal (MuMETAL, Magnetic Shield Corporation, Bensenville, IL) and copper layering and engineered with Mu-metal 18-inch channels to place sensors and EMF signal generator (BS-1000, Quasar Federal Systems, San Diego, CA) were built to allow for EMF recording at bedside instead of bringing the patient to a room designed for EMF recordings. The sensors and EMF signal generators were placed in a specific configuration. In this paper, each sensor’s spanning region is clarified in parentheses, for example, sensor 7 (left frontal lobe). The sensors were placed in the channels 9 inches away from the scalp, providing a 6.37-degree field of view. The known spatial relationship between the sensors allowed for identifying regions of overlap or opposite configurations, where sensors in opposing positions (180 degrees from each other) were expected to demonstrate opposite polarities for a specific EMF. Each sensor was also positioned with the positive end oriented toward the scalp.

Electromagnetic field (EMF) data collection and analysis

Detailed methodology is described in previous studies [9, 12-13]. Once localization of brain injuries with corresponding frequency selection was achieved, specific EMF stimulation tailored to each patient for modulation of the neuronal activities and the associated frequencies was delivered. An EMF signal generator (BS-1000, Quasar Federal Systems, San Diego, California) was used to deliver various voltage stimulations at the targeted frequency of interest (FOI). The optimal voltage stimulation was achieved when the EMF patterns of the sensor of interest (SOI) became a peak or at a similar amplitude as that of its opposing sensor (OS) Simultaneously, after each escalating dosage of voltage stimulation, clinical assessment was performed to evaluate for improvement in clinical symptoms or deficits and evaluation of the post-stimulation EMF pattern.

After optimal voltage stimulation was achieved to reverse abnormal EMF patterns to normal EMF patterns, if the patient still endorsed clinical symptoms or deficits, then the patient underwent incremental increases in duration of continuous EMF stimulation until improvement or resolution of clinical symptoms or deficits, and the sustained EMF change was attained [9]. Based on the previous animal study, the starting duration of EMF stimulation was 3 minutes [14]. The following regime was used: 3 minutes, 5 minutes, 8 minutes, and 10 minutes. During and after each increase in duration of a tailored and optimal EMF stimulation, patients were asked or examined regarding their neurological symptoms or deficits, and post-stimulation EMF data were also analyzed to evaluate for sustained changes in amplitude.

## Results

Ten patients were included in this study with a mean age of 47.1 years (range 23 years to 81 years). Mechanisms of injury included spontaneous hypertensive intracranial hemorrhage (1 patient) and head trauma after motor vehicle collision, dirt bike accident, and ground-level fall (9 patients). Radiographic findings included spontaneous basal ganglia hemorrhage (1 patient), isolated traumatic subdural hematoma (1 patient), traumatic subarachnoid hemorrhage (1 patient), and no intracranial abnormalities (7 patients). The patients’ brain EMF was detected over 0.5 Hz to 12 Hz from each of the 20 sensors at 5000 times per second. The brain EMF was localized, analyzed for normal and abnormal signals, and the abnormal EMF signal corresponding to the clinical pathology was treated with prescribed EMF stimulation. Clinical resolution of their neurological symptoms or remaining asymptomatic was achieved in five patients after three minutes of continuous EMF stimulation, two patients after five minutes of continuous EMF stimulation, and one patient after 10 minutes of continuous EMF stimulation (Table 1). Patient 8 declined to continue with the study after three minutes of continuous EMF stimulation, and patient 9 declined to continue with the study after five minutes of continuous EMF stimulation. Neither of these patients had abnormal or unusual effects or sensations.

**Table 1:** Electromagnetic field stimulation Hz: Hertz; V: Voltage; SOI: sensor of interest; OS: opposing sensor

Patient	Targeted Frequency of Interest	Voltage (V) Applied	Duration in Minutes	Clinical Assessment After Electromagnetic Field Stimulation	Post-electromagnetic Field Stimulation Interpretations
1	8.3 Hz	1.0 V	3 minutes	After 3 minutes, the patient remained neurologically asymptomatic.	After 3 minutes, SOI showed a peak and was at a higher amplitude than OS.
2	8.6 Hz	1.0 V	3 minutes	After 3 minutes, the patient reported resolution of the headache from the initial 2/10 pain.	After 3 minutes, SOI showed a similar amplitude to OS.
3	7.7 Hz	3.0 V	3 minutes	After 3 minutes, patient reported headache of 1/10 pain.	After 3 minutes, SOI showed a peak and had a similar amplitude to OS.
			5 minutes	After 5 minutes, patient reported resolution of headache from the initial 5/10 pain.	After 5 minutes, SOI was at a higher amplitude than OS.
4	7.3 Hz	3.0 V	3 minutes	After 3 minutes, patient remained neurologically asymptomatic.	After 3 minutes, SOI showed a peak.
5	7.6 Hz	8.0 V	3 minutes	After 3 minutes, patient reported resolution of headache from the initial 1/10 pain.	After 3 minutes, SOI showed a peak.
6	7.9 Hz	10.0 V	3 minutes	After 3 minutes, patient reported headache of 2/10 pain.	After 3 minutes, SOI had a similar amplitude as OS.
			5 minutes	After 5 minutes, patient reported resolution of headache from the initial 8/10 pain.	After 5 minutes, SOI showed a peak and was at a higher amplitude than OS.
7	8.7 Hz	10.0 V	3 minutes	After 3 minutes, patient reported headache of 5/10 pain.	After 3 minutes, SOI showed a positive slope and was at a lower amplitude than that of OS.
			5 minutes	After 5 minutes, patient reported headache of 3/10 pain.	After 5 minutes, SOI showed a plateau and was at a lower amplitude than OS.
			8 minutes	After 8 minutes, patient reported headache of 2/10 pain.	After 8 minutes, SOI showed a plateau and was at a lower amplitude than OS.
			10 minutes	After 10 minutes, patient reported resolution of headache from the initial headache of 10/10 pain.	After 10 minutes, SOI showed a positive slope and was at a lower amplitude than OS.
8	9.5 Hz	10.0 V	3 minutes	After 3 minutes, patient reported headache of 3/10 pain.	After 3 minutes, SOI remained at a valley and was at a lower amplitude than OS. (Patient declined to continue with the study.)
9	5.2 Hz	3.0 V	3 minutes	After 3 minutes, patient still endorsed expressive aphasia and right upper arm weakness.	After 3 minutes, SOI became a peak and was at a lower amplitude than OS.
			5 minutes	After 5 minutes, patient reported improvement in expressive aphasia and right upper arm weakness. (Patient declined to continue with the study.)	After 5 minutes, SOI showed a negative slope and was at a lower amplitude than OS. (Patient declined to continue with the study.)
10	10.4 Hz	10.0 V	3 minutes	After 3 minutes, patient reported resolution of headache from the initial 7/10 pain.	After 3 minutes, SOI showed a negative slope and was at a similar amplitude as OS.

Here, the authors use patient 6 as an illustrated example.

**Figure 1:**
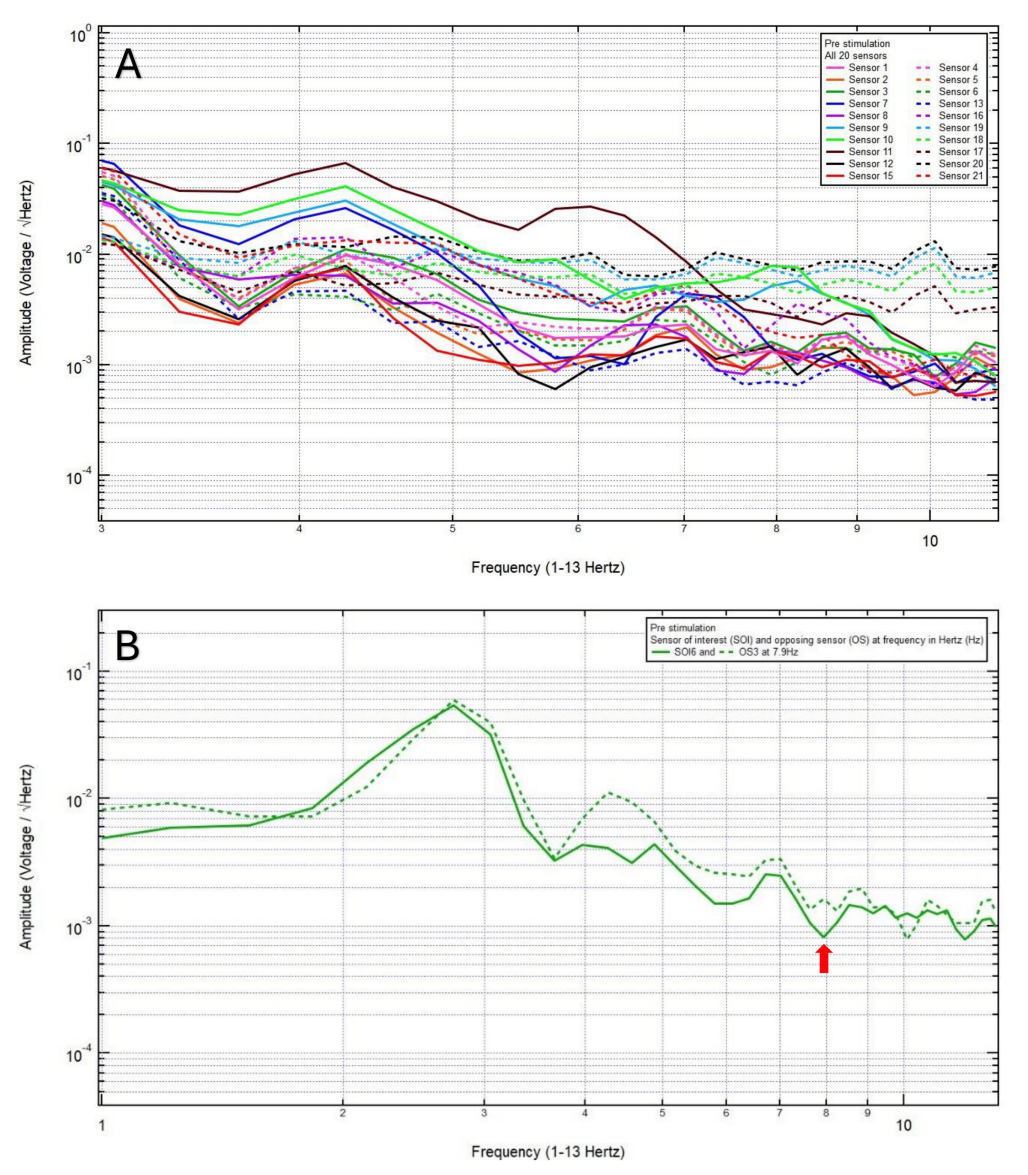
The patient’s pre-stimulation EMF data showed localization of the site of brain injury to sensor of interest 6 (left motor cortex and deeper structures) and opposing sensor 3 (right sensory cortex and deeper structures) (A) and revealed a pair of peak and valley at 7.9 Hz (red arrow) which was selected as the frequency of interest (B). EMF: Electromagnetic field. [12-13]

**Figure 2:**
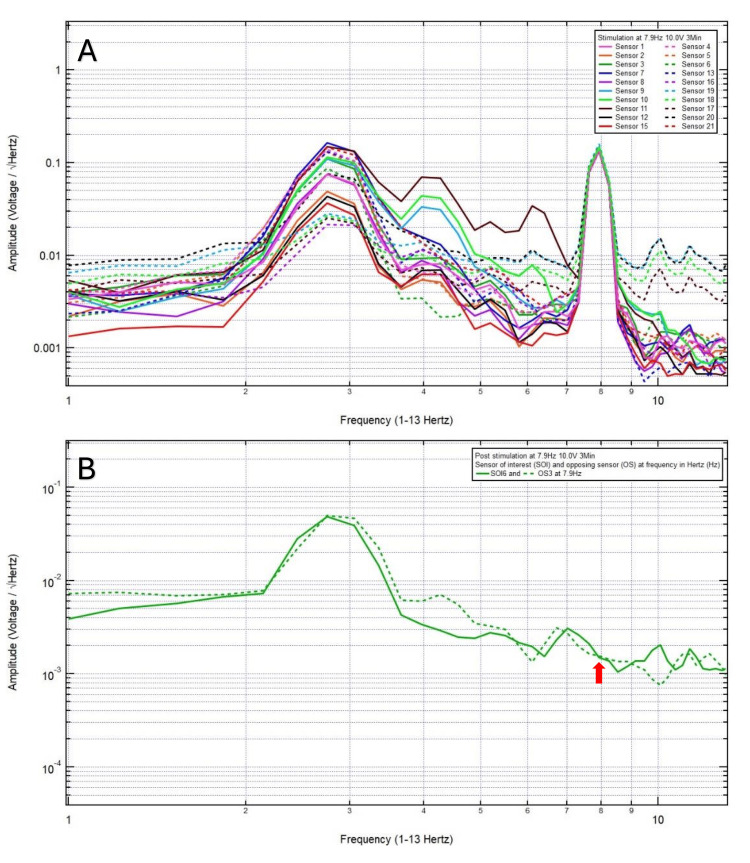
Continuous electromagnetic field stimulation at 7.9 Hz 10.0 V over 3 minutes was applied (A). Post stimulation electromagnetic field showed similar amplitude (red arrow) between the sensor of interest 6 and the opposing sensor 3 (B). Post stimulation, the patient reported headache of 2/10.

**Figure 3:**
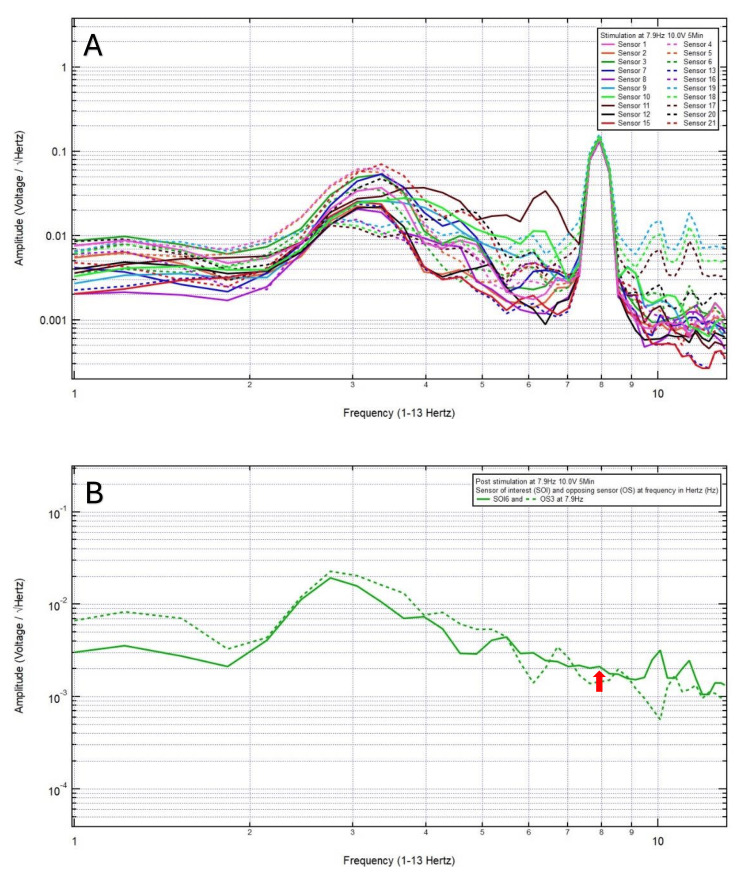
Continuous electromagnetic field stimulation at 7.9 Hz, 10.0 V over 5 minutes was delivered (A). Post-stimulation electromagnetic field showed the sensor of interest 6 was at a peak and higher amplitude than that of the opposing sensor 3 (B). Post-stimulation, the patient reported resolution of his headache from an initial headache of 8/10.

A 46-year-old left-handed male presented with a chief complaint of left vertex headache, 8/10 pain, after a motor vehicle collision, with head strike and loss of consciousness. The patient was GCS15 and had left eye ecchymosis and facial swelling. The patient’s pre-stimulation EMF data showed localization of the site of brain injury to SOI6 (left motor cortex and deeper structures) and OS3 (right sensory cortex and deeper structures) (Figure 1A) and revealed a pair of peak and valley at 7.9 Hz (red arrow) which was selected as the FOI (Figure 1B). Continuous EMF stimulation at 7.9 Hz, 10.0 V over 3 minutes was applied (Figure 2A). Post-stimulation, the patient reported a headache improved to 2/10 pain, and the post-stimulation EMF showed similar amplitude (red arrow) between SOI and OS (Figure 2B). Given that the patient still endorsed a headache, and post-stimulation EMF recording and assessment did not demonstrate a sustained change, a longer duration of continuous EMF stimulation was delivered, aiming to achieve resolution of his headache. Therefore, continuous EMF stimulation at 7.9 Hz, 10.0 V over 5 minutes was delivered (Figure 3A). Post-stimulation, the patient reported resolution of his headache from an initial headache of 8/10. Post-stimulation EMF showed the SOI was at a peak and higher amplitude than that of the OS (Figure 3B). Given the complete resolution of his headache, there was no indication to increase the duration of stimulation to 8 minutes. 

## Discussion

In this prospective study, real-time feedback from patients with atraumatic and traumatic brain injury (TBI) regarding their symptoms or deficits and the recording and assessment of the brain EMF guided the treatment protocol in regard to whether or not to increase the duration of continuous EMF stimulation. This study found that an incremental increase in duration of continuous EMF stimulation in patients with atraumatic and TBI was correlated with sustained EMF pattern changes and improvement or eventual resolution of neurological symptoms and deficits. Previously, the authors investigated techniques for the utilization of optimal voltage for continuous EMF stimulation whereby the abnormal EMF patterns of sensors of interest were transformed into peaks or similar amplitudes as those of the opposing sensors [9]. In five patients, these EMF changes at optimal voltage of 3-minute stimulation correlated with the resolution of their neurological symptoms, or they remained asymptomatic and sustained brain EMF changes. On the other hand, the three patients out of five who agreed to continue with the study reported resolution of their headache after a longer duration of continuous EMF stimulation, with their post-stimulation EMF patterns with either peak, higher amplitude, or positive slope. This aligns with the authors’ hypothesis that longer duration of stimulation should induce higher amplitude and correlate with improvement in clinical symptoms or deficits and sustained brain EMF changes. If patient 9 had continued with a longer duration of continuous EMF stimulation (e.g., 8 minutes), the authors speculate further improvement which may have even crossed over to the more significant neurological deficits of expressive aphasia and right upper arm weakness or possibly resolution of his deficits as this patient had improvement in his aphasia and symptoms with shorter durations of treatment. The families of the remaining two patients objected to the patients’ wishes for continued treatment to continue longer stimulation. There were no unusual events that occurred during any treatment. Further studies will be needed to identify those patients who can be identified early as needing extended treatment and to treat them. It is hypothesized that utilizing continuous EMF stimulation is required to treat more significant deficits. There is no evidence to date that extended treatments cause short-term or long-term complications, but this is yet to be studied in the laboratory.

Unlike other technologies such as PEMF, ELF-MF, and magnetoencephalography, the portable helmet, its sensors, and stimulator allow for easy transportation to the patient’s room, provides real-time feedback throughout the treatment course, precisely targets treatment, allows for treatment stimulation adjustments in real-time, and improve neurological symptoms and deficits in one session instead of over days in a single novel device [1-8]. The authors have also performed and observed improvement in sustained devastating neurological symptoms and deficits in patients treated with a longer duration of stimulation, 15-30 minutes.

The observation that longer duration of stimulation correlates with clinical improvement or resolution of symptoms and sustained EMF changes, and was required in more severely damaged patients, suggests perhaps that the extra stimulation induces more molecular and cellular changes. The measurement of each area of the brain under the sensor and its corresponding functions can be quantified from 0.001 Hz to 2,500 Hz with the current equipment and techniques [9-15]. The measured brain EMF corresponds to other functions of thought, consciousness, neuronal circuits, cell activity, cell function, genetic, and cell transcription in the normal and diseased brain [16-23]. For example, less neuronal apoptosis was found in a swine that underwent immediate EMF stimulation post TBI when compared to the control swine (without EMF stimulation) and the swine with delayed EMF stimulation at 48 hours post TBI and higher levels of neuron-specific enolase, indicating increased histologic viability, in swine that received EMF stimulation [18]. Several genes were differentially expressed in a pilot study using the Yucatan miniature swine model of TBI with and without 3-minute EMF stimulation: INSC, TTR, CFAP126, SEMA3F, CALB1, CDH19, and SERPINE1. These genes were associated with cell proliferation, cell migration, immune cell infiltration, thyroid hormone transportation, myelination, and reactive oxygen species regulation [20]. While the range of unique EMF corresponds to macroscopic and microscopic functions, the vast majority have yet to be qualified and quantified because most brain diseases have yet to be studied.

Overall, this study reveals novel technology of real-time measurement of brain EMF, the ability to adjust stimulation parameters based upon real-time measurements, and continuous EMF stimulation of longer duration at the optimal voltage and frequency [9, 12-13]. The range of brain EMF in normal and diseased human functions can be measured to precise brain areas in a non-invasive, non-contact fashion from a distance using this lightweight portable equipment that can be donned in hospitals, clinics, ambulance, or other place, and as far as 63 cm away [9-13, 24-27]. When using this new technology, patients with atraumatic and traumatic brain injuries can be diagnosed based on their EMF. Then, prescribed targeted EMF stimulation can be applied and adjusted in real-time in order to lead to improvement or resolution of clinical symptoms or deficits and sustained EMF changes. Thus, this finding illustrates the possibility of real-time EMF evaluation and the ability to target and modulate neuronal signaling through EMF stimulation. It further summarizes the progress to date and indicates the efficacy of tailored and precise EMF stimulation to the specific patient, to the specific location of abnormality, and for a specific pathology in a sample of traumatic and atraumatic patients, including concussion [9, 12-13]. Further studies can be conducted to evaluate long-term effects and treatments for additional neurological disease states, and evaluate treatment and outcomes for those with more severe disease states.

Limitations

The small sample size and a lack of long-term follow-up limit this study’s ability to generalize the findings and show the long-term effects of EMF modulation. Also, a placebo effect may induce clinical changes even though the EMF abnormalities were mitigated. Moreover, this study did not use objective metrics. Larger sample sizes, long-term follow-ups, and objective metrics can aid in modifying and improving the current protocol for EMF modulation in patients with atraumatic and traumatic brain injuries.

## Conclusions

This study reveals novel technology for real-time measurement of normal and abnormal brain EMF and a prescribed tailored continuous EMF stimulation of longer duration at the optimal voltage and frequency based upon real-time feedback in a sample of patients with traumatic and atraumatic brain conditions. It illustrates the progress made to date, necessitating real-time evaluation and adjustment of brain EMF for EMF stimulation. It further indicates the efficacy of tailored and precise EMF stimulation to the specific patient, for a specific location, and for a specific pathology. When performed using this new technology, these patients studied with atraumatic and traumatic brain injuries can obtain improvement or resolution of clinical symptoms or deficits and sustain EMF changes. The range of unique brain EMF changes has been shown to correspond to macroscopic and microscopic functions, the vast majority of which have yet to be identified, qualified, and quantified. This technology has performed EMF measurements in real-time using the non-invasive, lightweight helmet, and with continuous feedback, the range of brain EMF has been stimulated at the optimal frequency and voltage with or without longer duration of stimulation in a precise and prescribed manner to produce sustained genetic and neuronal changes to improve, recover, and enhance the brain function in patients and mini-swine studied with atraumatic and TBI and improve or resolve neurological symptoms or deficits. This technology is in its infancy, and much extensive research is needed to further refine optimal treatment parameters for short-term and long-term clinical improvements and symptom relief in patients with the vast majority of neurological diseases.
